# Supplementary material for: Primary care physicians’ knowledge of travel vaccine and malaria chemoprophylaxis and associated predictors in Qatar
Source: PLoS One. 2022 Mar 31;17(3):e0265953. doi: 10.1371/journal.pone.0265953 (PMC8970385; doi:10.1371/journal.pone.0265953)
Supplement: S1 File — (PDF) [file pone.0265953.s001.pdf]

Appendix

Study Questionnaire

" Primary Care Physicians' Knowledge of Travel Vaccine and Malaria Chemoprophylaxis and Associated Predictors in Qatar"

Sociodemographic and practice-related characteristics

Q #	Question	Responses	
1	**Age**	❑ (In completed years) ________________	
2	**Gender**	❑ Male	❑ Female
3	**Country of your medical degree**	❑ ______________________________	
4	**Total number of years in general practice**	❑ (In completed years) ________________	
5	**Frequency of pretravel consultations in the last 6 months**	❑ Do not counsel travellers at all<br>❑ <10 consultations/ month	❑ ≥10 consultations/ month
6	**Do you have previous postgraduate experience in tropical medicine/ developing countries** (any engagement in travel medicine practice after graduation from medical school).	❑ No<br>❑ Yes (if yes, for how long?) …………………year(s)	
7	**Do you have previous postgraduate training in travel medicine or related area** (e.g. tropical medicine)?	❑ No	❑ Yes
7.1	**Type**(s) **of training received?**<br>(select all applicable)	❑ Workshop<br>❑ Certified short course<br>❑ Membership/Fellowship of travel medicine related professional organization	❑ Diploma<br>❑ Master degree<br>❑ Others, please specify ____________________

Sources of travel medicine information (to deliver relevant service)

**How often do you use the followings as a source for travel medicine information?**					
		**Every time**	**Often**	**Rarely**	**Never**
**8**	**Internet websites** (e.g.: Travax, CDC, WHO)	❑	❑	❑	❑
**9**	**Specialized textbooks**	❑	❑	❑	❑
**10**	**Specialized journals**	❑	❑	❑	❑
**11**	**Other colleagues**	❑	❑	❑	❑
**12**	**Personal travel experience**	❑	❑	❑	❑
**13**	**Others**, please specify____________________	❑	❑	❑	❑

Knowledge in Travel medicine

**A. Are the following vaccines indicated for <u>MOST</u> adult travelers** (previously unvaccinated) **going to the following destinations?** Please select the most appropriate answer for each destination					
**14**	**Kenya** (East Africa)	**Cholera**	❑ Yes	❑ No	❑ I don't know
**15**		**Hepatitis A**	❑ Yes	❑ No	❑ I don't know
**16**		**Typhoid**	❑ Yes	❑ No	❑ I don't know
**17**		**Rabies**	❑ Yes	❑ No	❑ I don't know
**18**	**Saudi Arabia for Hajj**	**Pneumococcal**	❑ Yes	❑ No	❑ I don't know
**19**		**Seasonal flu**	❑ Yes	❑ No	❑ I don't know
**20**		**Meningococcal**	❑ Yes	❑ No	❑ I don't know
**21**		**Dengue fever**	❑ Yes	❑ No	❑ I don't know
**22**	**Thailand** (South East Asia)	**Cholera**	❑ Yes	❑ No	❑ I don't know
**23**		**Hepatitis A**	❑ Yes	❑ No	❑ I don't know
**24**		**Typhoid**	❑ Yes	❑ No	❑ I don't know
**25**		**Yellow fever**	❑ Yes	❑ No	❑ I don't know

**B. Which of the following destinations require the use of anti-malarial chemoprophylaxis for MOST adult travelers?** Please select the most appropriate answer for each destination				
**26**	**Rural Thailand**	❑ Yes	❑ No	❑ I don't know
**27**	**Turkey**	❑ Yes	❑ No	❑ I don't know
**28**	**Tanzania**	❑ Yes	❑ No	❑ I don't know
**29**	**Sri Lanka**	❑ Yes	❑ No	❑ I don't know

THANK YOU FOR YOUR PARTICIPATION IN THIS STUDY
